# An assessment of residents’ and fellows’ personal finance literacy: an unmet medical education need

**DOI:** 10.5116/ijme.5918.ad11

**Published:** 2017-05-29

**Authors:** Fahd A. Ahmad, Andrew J. White, Katherine M. Hiller, Richard Amini, Donna B. Jeffe

**Affiliations:** 1 Washington University School of Medicine, St. Louis, Missouri, USA; 2 University of Arizona College of Medicine, Tucson, Arizona, USA

**Keywords:** Personal finance, graduate medical education, financial literacy, debt

## Abstract

**Objectives:**

This study aimed to assess residents’ and fellows’ knowledge of finance principles that may affect their personal financial health.

**Methods:**

A cross-sectional, anonymous, web-based survey was administered to a convenience sample of residents and fellows at two academic medical centers.  Respondents answered 20 questions on personal finance and 28 questions about their own financial planning, attitudes, and debt. Questions regarding satisfaction with one’s financial condition and investment-risk tolerance used a 10-point Likert scale (1=lowest, 10=highest).  Of 2,010 trainees, 422 (21%) responded (median age 30 years; interquartile range, 28-33).

**Results:**

The mean quiz score was 52.0% (SD = 19.1). Of 299 (71%) respondents with student loan debt, 144 (48%) owed over $200,000.  Many respondents had other debt, including 86 (21%) with credit card debt. Of 262 respondents with retirement savings, 142 (52%) had saved less than $25,000. Respondents’ mean satisfaction with their current personal financial condition was 4.8 (SD = 2.5) and investment-risk tolerance was 5.3 (SD = 2.3). Indebted trainees reported lower satisfaction than trainees without debt (4.4 vs. 6.2, F _(1,419)_ = 41.57, p < .001).   Knowledge was moderately correlated with investment-risk tolerance (r=0.41, p < .001), and weakly correlated with satisfaction with financial status (r=0.23, p < .001).

**Conclusions:**

Residents and fellows had low financial literacy and investment-risk tolerance, high debt, and deficits in their financial preparedness.  Adding personal financial education to the medical education curriculum would benefit trainees.  Providing education in areas such as budgeting, estate planning, investment strategies, and retirement planning early in training can offer significant long-term benefits.

## Introduction

Physicians in the United States earn high salaries after training however they enter the workforce several years after and with greater debt than peers with other professional graduate degrees. The median debt of US medical school graduates is higher than any other post-graduate training population,^1^ with 82% of recent graduates carrying over $100,000 in medical student loans.^2^^,^^3^ Trainees who choose fellowship training after residency extend their time working at lower income levels while increasing their debt burden.^4^  Fewer physicians in the US are choosing self-employment after training,^5^ and these physicians may not perceive a need for learning basic finance or business principles, jeopardizing their future financial health. Financial literacy is not a requirement to be a competent physician, but can significantly impact job satisfaction and productivity.^6^ In most other countries, physicians are among the most well-educated and well-compensated professionals, even though their future incomes and levels of commensurate debt may be lower compared to physicians in the US. Yet, even in countries with different physician reimbursement systems, educating medical trainees regarding budgeting, savings, and financial planning could provide significant long-term benefits for individuals and their medical practice. Despite this, few studies have evaluated the financial literacy of medical trainees, or evaluated the efficacy of interventions to improve financial literacy and changes in behavior.  The primary objective of this study was to conduct a needs assessment of residents’ and fellows’ financial literacy (knowledge of personal-finance principles) and their financial status. This information can be used to design educational interventions that focus on improving medical trainees’ financial literacy.

## Methods

### Study design

A cross-sectional anonymous survey was administered to a convenience sample of residents and fellows at two US academic medical centers: Washington University School of Medicine in St. Louis and University of Arizona College of Medicine.

### Study participants

The Institutional Review Boards at both institutions approved this study as minimal risk and exempt from ongoing review.  All residents and fellows at each study site were invited to participate in the survey via email with a link to the online survey sent through the graduate medical education office at each institution and from residency/fellowship program directors.  All responses were anonymous.  Three follow-up reminders were sent, and responses were collected from January-March 2015 at Washington University and from June-August 2015 at University of Arizona College of Medicine. The survey was programmed and the data collected using the online Research Electronic Data Capture (REDCap) software. Respondents were eligible to win one of 40 $25 Amazon.com gift cards from Washington University or 25 gift cards at University of Arizona. Respondents’ contact information for the lottery was not linked to survey responses. 

Recruitment emails were sent to 1,114 trainees at Washington University and 896 trainees at University of Arizona College of Medicine.  The graduate medical education office at Washington University sent invitation emails to all trainees, while individual program directors at the University of Arizona sent the email invitations to their trainees.   Some trainees at the University of Arizona started after or completed training during their enrollment period and might not have received all email invitations/reminders.  Nevertheless, all trainees were included when determining the response rate as we could not verify which trainees received which emails.  At Washington University, there were 289 responses (26%), and at University of Arizona, there were 133 responses (15%), for a total of 422 responses (21%). 

 Table 1 provides characteristics of respondents by institution.  Respondents from the two sites were similar with regard to age, marital status, dependent children, and parental education levels.  A higher percentage of respondents from Washington University indicated their future work plans would be in an academic or university environment.

**Table 1:** Characteristics of resident and fellow participants (N=422)

Demographics	Washington University (n=289)	University of Arizona (n=133)	Total (N=422)
Age in years, median (interquartile range, IQR)	30 (28-33)	30 (28-33)	30 (28-33)
Post-graduate year level, median (IQR)	3 (2-5)	2 (1-4)	3 (2-4)
Current Marital Status, n (%)			
Married/Partner	174 (60.2)	74 (55.6)	248 (58.8)
Single	108 (37.4)	54 (40.6)	162 (38.4)
Separated	1 (0.3)	0	1 (0.2)
Divorced	2 (0.7)	1 (0.8)	3 (0.7)
Did not answer	4 (1.4)	4 (3.0)	8 (1.9)
Have dependent children, n (%)			
Yes	89 (30.8)	33 (24.8)	122 (28.9)
No	200 (69.2)	100 (75.2)	300 (71.1)
Highest maternal education, n (%)			
Some high school or high school graduate	29 (10.0)	9 (6.8)	38 (9.0)
Specialized business/technical training	4 (1.4)	4 (3.0)	8 (1.9)
Some college or college degree	114 (39.4)	66 (49.6)	180 (42.7)
Some graduate school or graduate degree(s)	137 (47.4)	51 (38.3)	188 (44.5)
Did not answer	5 (1.7)	3 (2.3)	8 (1.9)
Highest paternal education			
Some high school or high school graduate	21 (7.3)	9 (6.8)	30 (7.1)
Specialized business/technical training	3 (1.0)	5 (3.8)	8 (1.9)
Some college or college degree	83 (28.7)	43 (32.4)	126 (29.9)
Some graduate school or graduate degree(s)	177 (61.2)	72 (54.1)	249 (59.0)
Did not answer	5 (1.7)	4 (3.0)	9 (2.1)
Future work plans			
Academic/university	121 (41.9)	31 (23.3)	152 (36.0)
Private	70 (24.2)	41 (30.8)	111 (26.3)
Uncertain	95 (32.9)	57 (42.9)	152 (36.0)
Other	3 (1.0)	3 (2.3)	6 (1.4)
Did not answer	0	1 (0.8)	1 (0.2)
Specialty			
Internal Medicine	62 (21.5)	35 (26.3)	97 (23.0)
Pediatrics	61 (21.1)	17 (12.8)	78 (18.5)
Emergency Medicine	18 (6.2)	21 (15.8)	39 (9.2)
Radiology	25 (8.7)	12 (9.0)	37 (8.8)
Surgery	28 (9.7)	6 (4.5)	34 (8.1)
Anesthesiology	27 (9.3)	2 (1.5)	29 (6.9)
Psychiatry	12 (4.2)	11 (8.3)	23 (5.5)
Pathology	9 (3.1)	9 (6.8)	18 (4.3)
Other	43 (14.9)	13 (9.8)	56 (13.3)
Did not answer	4 (1.4)	7 (5.3)	11 (2.6)

### Data collection

A 48-item questionnaire on personal finance and investment knowledge was derived largely from existing national surveys from the Financial Industry Regulatory Authority (FINRA), with their permission.^7^^-^^10^ FINRA is an independent, not-for-profit organization, authorized by the US government to create and enforce rules governing financial brokers that create protections for individual investors. As part of this mission, they periodically assess the financial knowledge of US citizens. FINRA created these survey instruments in conjunction with experts in finance and business, including members of the Employee Benefit Research Institute and the American Institute of Certified Public Accountants. Survey questions were piloted by FINRA using multiple in-person interviews, then they were further modified after obtaining feedback from delivering the questions using Computer Aided Telephone Interviews.  After reviewing FINRA questionnaires ourselves, we consulted a physician expert in personal finance to provide additional review of the FINRA questions and to develop additional questions to answer our study aims.  All questions were then reviewed for accuracy and clarity of wording by physicians not on the study team before distribution.

The questionnaire (Appendix) included multiple-choice and open-ended questions in two parts: 1) twenty questions assessing knowledge of basic financial topics regarding savings and investing, and 2) twenty-eight questions assessing attitudes and behaviors about trainees’ own personal financial status and planning, investment-risk tolerance, and satisfaction with their financial condition.  Questions regarding satisfaction with one’s financial condition and investment-risk tolerance used a 10-point Likert scale (1=lowest, 10=highest). 

### Statistical analysis

Responses were included for analysis if respondents provided any answer to more than half of the 20 knowledge questions (including "I don't know") and answered any of the subsequent demographic questions. When calculating the proportion of correct responses to the knowledge questions, skipped questions and “I don’t know” responses were considered incorrect responses.

Chi-square or Fisher’s exact tests, as appropriate, were used to measure associations between two categorical variables (e.g., demographics, whether or not respondent had savings, or checked his/her credit report or score). Pearson correlations measured associations among the continuous variables (total knowledge scores, satisfaction with personal financial condition, and investment-risk tolerance), and one-way analyses of variance (ANOVA) measured between-groups differences in these continuous variables.  Analyses were performed with IBM SPSS Statistics version 22.0.

## Results

### Quiz scores

The Appendix provides the individual quiz questions, answer choices, and proportion of respondents answering each question correctly. The mean quiz score (percentage of 20 items answered correctly) was 52% (SD = 19.1; range 0-100%).  Some questions relating to basic investing principles had a relatively low proportion of correct responses, e.g., that no-load mutual funds carry no sales charges (13%) and that if interest rates rise, bond prices tend to fall (19.9%).  A greater proportion of respondents with children (76/121 [62.8%]) than respondents without children (120/300 [40.0%]) knew that a Section 529 Plan (in the US) is a tax-advantaged way to provide savings for college (χ2 (3, N=421) = 18.93, p < .001). 

### Savings, debt, and income

**Figure 1:**
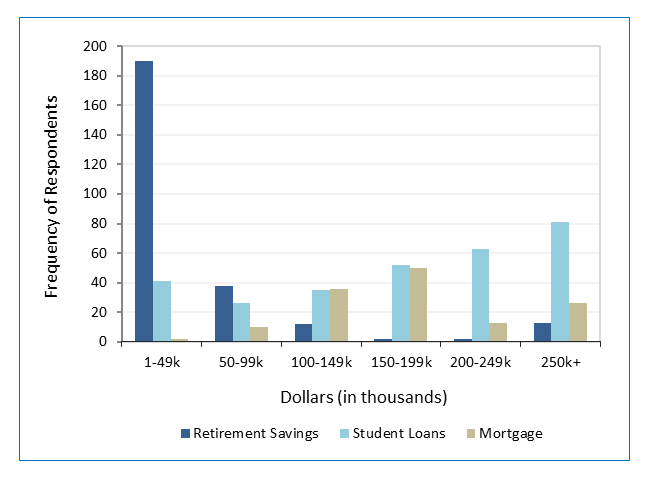
Amount of money in retirement savings, student loan debt, and mortgage debt among trainees who have savings or are indebted (N=387).  Only trainees with any retirement savings (n=262), student loans (n=299), or mortgages (n=137) are represented.

Table 2 provides information regarding savings, debt, and overall spending.  Nearly one-third of respondents indicated some difficulty in meeting monthly expenses. Respondents reported high debt levels, with 332 (79%) reporting some combination of mortgage, credit card, or student loan debt (Table 2 and Figure 1). One hundred forty-four (48% of 299) respondents with student loans owed over $200,000, and 126 (92% of 137) of those with a mortgage owed more than $100,000.  Eighty-six (20%) respondents had credit card debt that would not be paid off at the end of the month, 27 (32%) of whom expected to carry more than $10,000 to the next month.

Retirement savings were low, with 160 (38%) respondents reporting no retirement savings, and of 262 respondents with retirement savings, 56% (142/256) had under $25,000 (six participants did not provide savings amount).  Respondents who had projected their necessary retirement savings were more likely to have reported having some retirement savings than those who did not (83% [139/167] vs. 49% [123/253], χ2 (1, N=420) = 51.37, p < .001).  Respondents with any debt (credit card, mortgage, student loan) had higher mean quiz scores than those without debt (53.1 [SD = 18.2] vs. 48.0 [SD = 21.7], F _(1,419)_ = 5.11, p =0.02)

### Financial planning

Table 2 also provides an overview of respondents’ financial planning status.  One hundred ninety-seven had obtained professional financial advice in the previous five years, with 57.8% receiving advice for free, and the remainder paying for advice in various ways.  Although few respondents had wills, respondents with children were more likely to have a will than those without children (17/121 [14%] vs. 14/300 [5%], χ2 (1, N=421) = 11.13, p = .001). Many respondents had not checked their credit score or report, with 154 (37%) of all respondents checking neither in the past year.

### Satisfaction and investment-risk tolerance

Respondents’ mean willingness to take investment risk was 5.3 (SD = 2.3), with a bell-shaped distribution. Investment-risk tolerance did not differ significantly between respondents with and without debt (5.2 vs. 5.6, F _(1,418)_ = 2.19, p = .14).  Higher knowledge scores were moderately correlated with greater investment-risk tolerance (r=0.408, p < .001).

Respondents’ mean satisfaction with their personal financial condition was 4.8 (SD = 2.5), with nearly all respondents answering between 1-8.  Trainees with any debt (student loan, mortgage, or credit card) reported lower satisfaction than trainees without debt (4.4 vs. 6.2, F _(1,419)_ = 41.57, p < .001).  Higher knowledge scores on the quiz were weakly correlated with greater satisfaction of personal financial condition (r=.226, F_(__20,401)_ = 2.11, p < .001)

**Table 2:** Financial status and practices of trainees (N=422)

Survey questions	Survey responses n (%)
**Financial Planning Status**	
Checked credit score in prior 12 months	243 (57.6)
Obtained copy of credit report in prior 12 months	218 (51.7)
Asked financial professional for advice in past 5 years^*^	197 (46.7)
Types of financial advice obtained^†^	
Debt counselling	93 (47.2)
Savings or investments	136 (69.0)
Taking out a loan or mortgage	68 (34.5)
Insurance of any type	109 (55.3)
Tax planning	59 (30.0)
Estate planning	25 (12.7)
Asset protection	34 (17.3)
How paid for financial advice^†^	
Advice was free	114 (27.0)
Commission on purchased products	28 (6.6)
Yearly retainer	10 (2.4)
Asset under management (AUM) fee	10 (2.4)
Combination of above	22 (5.2)
Doesn't know	10 (2.4)
Attended financial planning seminars at university^*^	129 (30.6)
Have a will^*^	31 (7.3)
**Savings**	
Have an emergency fund for 3 month's expenses	278 (65.9)
Can come up with $2,000 within the next month	321 (76.1)
Have projected necessary retirement savings^*^	168 (39.8)
Have retirement savings (any)	262 (62.1)
Money set aside for children's college education (any)^*‡^	47 (38.5)
**Debt**	
Have a mortgage (any)^*^	137 (32.5)
Have credit card debt (any)^*^	86 (20.4)
Have student loans (any)^*^	299 (70.9)
Have any of the above debt^*^	332 (78.7)
**Income and expenses**	
Difficulty meeting monthly expenses	
Very difficult	24 (5.7)
Somewhat difficult	111 (26.3)
Not at all difficult	287 (68.0)
Money spent in relation to annual income over prior year	
Spent less than income	283 (67.1)
Spent about the same as income	89 (21.1)
Spent more than income	50 (11.8)
Confidence in covering an unexpected $2,000 expense	
Certain	321 (76.1)
Probably	71 (16.8)
Probably not	19 (4.5)
Definitely not	11 (2.6)

*Items with 1 missing response;

†Among those 197 respondents who obtained professional advice;

‡Among those 122 respondents with dependent children;

## Discussion

We found serious deficits in financial knowledge in a broad range of topics and in financial planning among trainees at two academic medical institutions in the US Trainees reported high debt levels, minimal retirement investments, and having difficulty with cash flow.  In addition, trainees reported low levels of satisfaction with their financial status and were largely averse to high-risk investments, which generally have potential for greater yield for future expenses, such as for their children’s education or their own retirement. Nearly one-third of respondents reported difficulty meeting their monthly expenses and reported spending all or more of their income each month.  Despite having an income similar to the national median income,^11^ and an expectation of a significantly higher future income after training, our study participants were less satisfied with their personal financial condition than the average US citizen.^9^ As educators, we cannot assume that knowledge deficits will be rectified with time; moreover, higher income does not necessarily come with greater financial knowledge.  There is increasing recognition that financial literacy offered at the workplace can improve workplace satisfaction and job performance, in addition to improving one’s financial health.^6^^,^^12^^-^^15^  There is every reason to expect that physicians would also benefit from such instruction.

Respondents had similar levels of mortgage debt, substantially higher student loan debt, and lower levels of credit card debt than the average US household.^16^  While lower than the national average, the prevalence of credit card debt was concerning, given the high interest rates,^17^ potential for creating emotional stress,^18^ and already high levels of student loan debt.  Reducing or eliminating credit card debt should be a high priority for medical students and graduates, as they face larger student debt burdens than other professional graduates.^1^

Fewer than half of the respondents had projected their necessary retirement savings or obtained professional advice from financial planners during the previous five years. While retirement savings are expected to be low early in post-graduate training, financial planning strategies to minimize additional debt and to build savings should already be in place. Many physicians in training care for seriously ill or dying patients and are taught about advance directives. However, most respondents, including those with children, lacked a will, a basic estate-planning tool. Only half of respondents had reviewed their credit report or credit score in the past year. Checking one’s credit report online is free up to three times per year,^19^ and checking one’s credit scores is available online without cost.^20^  Maintaining a good credit history is an important part of financial planning. 

Trainees also showed low levels of knowledge about health insurance. Few respondents knew that individuals must have a high-deductible health plan in order to make contributions to a HSA. Familiarity with different health insurance plans is important for healthcare providers so that they understand the requirements for, and ramifications of, different types of coverage, both personally and professionally for patient care.

As personal finance has been largely neglected by the medical education system, it has created a vacuum that has been filled by a cottage industry of financial advisors who exist solely to provide financial advice to physicians. Many advisors are highly qualified and well-intentioned, however some charge unreasonably high fees for their services, or encourage customers to buy inappropriate products. A recent study found that 20% of financial advisors have been disciplined for misconduct,^21^ and physicians in training are at high-risk for being the target (and victim) of unscrupulous advisors.  Medical educators have the opportunity to help physicians develop a financial knowledge base necessary to understand basic finance and investment principles. With improved financial literacy, physicians in training might better manage their debt and learn more about the benefits and risks of a variety of investment options, improve their current and future financial health, and be empowered to identify qualified and competent financial advisors.

Coursework about the basics of personal financial planning and important financial considerations relevant to medical practice and health insurance should be incorporated into the curriculum during medical school and graduate medical education.  Even brief interventions early in training, such as improving the understanding of the risk involved in different types of investments, or diversifying investments in order to minimize risk of loss of capital, could lead to large financial benefits.  Student loan debt is already very high for many trainees, and poor financial habits (e.g., misuse of credit cards) may add to their already stressed debt burden. Thus, financial education for medical trainees could show long-term improvements in physicians’ financial health, both personally and in practice.

The Accreditation Council for Graduate Medical Education (ACGME) has instituted duty-hour restrictions to reduce fatigue and help trainees perform at high levels during their clinical training.^22^  Financial stress also has the potential to negatively impact work performance during and after training.^23^^,^^24^  Educating medical students and trainees to help mitigate this stress should therefore be made an educational priority, and the Association for American Medical Colleges offers some financial planning resources for use.^25^  However, based on our needs assessment, we recommend, at a minimum, that every trainee receive specific instruction in the following aspects of financial education during medical school and residency:

1.     How to make a monthly budget

2.     Debt/loan management and credit scores/reports

3.     Savings and retirement planning options

4.     Life, health, and disability insurance

5.      Estate planning strategies

Several small, single-site studies have described assessment of financial knowledge or implementation of financial education for healthcare trainees^26^^-^^31^ Dhaliwal and colleagues found that a short, 90-minute seminar on personal finance led to a change in the types of investment chosen for medical residents’ retirement funds, demonstrating the potential for considerable impact from small interventions.^32^  Financial-literacy interventions in other fields have demonstrated the ability to improve financial decision-making and perceived well-being.^33^

Mizell and colleagues described a comprehensive financial-management curriculum for surgery residents at their institution.^26^ The curriculum covered an array of topics, with many lectures integrated into their mandatory Grand Rounds sessions.  The development and successful integration of this curriculum into an already demanding general surgery residency program demonstrates the potential for incorporating financial education into any medical or surgical training program.  This program benefited directly from the availability of a surgeon who is also a certified financial planner.  Whether such a curriculum could be broadly implemented in other training programs remains to be seen. 

We observed important deficits in core knowledge about personal finance topics, such as debt management, savings, and estate planning, which can have an immediate and long-term impact on one’s financial health.  Training on practice management, coding, and billing is also important for physicians, but content might vary across specialties and practice settings.  Teaching universal financial literacy skills in the workplace is increasingly common across a variety of industries.  With increasing levels of medical school debt, residents and fellows face critical financial challenges, some of which they might not even be aware, as they continue their post-graduate training.  Given the potential for negative consequences of financial stress on physicians’ emotional well-being and work performance,^23^^,^^24^ incorporating basic financial education into the curriculum during medical school and post-graduate training makes good professional sense.

### Limitations

A selection bias due to relatively low response rates and surveying a convenience sample of trainees at only two sites could limit the generalizability of our results. Although we only surveyed two institutions, the sample included a diverse sample of graduates from various medical schools. Moreover, personal finance education was not offered as part of the training curricula at either of these institutions and is not widely offered in medical training programs across the US. Thus, although participants might have been more interested in the topic than non-participants, we would not expect that participants differed greatly in terms of their financial knowledge and preparedness compared with non-participants at these two institutions nor with trainees at other training programs.  Another limitation is that the two sites enrolled at different times of year, which could have affected participation rates and participants’ responses.^34^ As with all self-reported attitudes and behaviors, participants’ responses could be subject to recall and self-protection bias.     

## Conclusions

The ACGME and medical educators at all levels of training should strive to improve the financial literacy of medical providers through implementation of personal finance, health insurance, and medical-practice-related financial curricula at US medical institutions.  Further, these curricular changes should be evaluated for their effectiveness in increasing physicians’ financial knowledge and engagement in financial-planning behaviors that could protect their financial security and reduce financial-related stress and its impact of work performance.  Future longitudinal studies should also evaluate changes in knowledge and behavior during and after training.

### Acknowledgments

The authors wish to thank James M. Dahle, MD, Department Chair, Utah Emergency Specialists, for his assistance in refining the survey tool, and Lauren Yaeger, MA, MLIS, St. Louis Children’s Hospital in partnership with Washington University School of Medicine in St. Louis, for her assistance in the literature search.

### Conflict of Interest

The authors declare that they have no conflict of interest.

## Supplementary material

Supplementary file 1 Appendix. Survey Questions
