# Supplementary material for: An assessment of residents’ and fellows’ personal finance literacy: an unmet medical education need
Source: Int J Med Educ. 2017 May 29;8:192–204. doi: 10.5116/ijme.5918.ad11 (PMC5457786; doi:10.5116/ijme.5918.ad11)
Supplement: Supplementary file 1 — Appendix. Survey Questions [file ijme-8-192-S1.pdf]

Appendix

Survey Questions

N=422

Part 1 - Financial Knowledge Quiz

Correct response bolded, with % of sample responding correctly shown for each question

All questions multiple-choice

1. Suppose you have $100 in a savings account earning 2 percent interest a year. After five years, how much would you have?
   - o **More than $102 (96.4%)**
   - o Exactly $102
   - o Less than $102
   - o I don't know
2. In general, investments that are riskier tend to provide higher returns over time than investments with less risk.
   - o **True (64.5%)**
   - o False
   - o I don't know
3. Buying a single company's stock usually provides a safer return than a stock mutual fund.
   - o True
   - o **False (87.2%)**
   - o I don't know
4. Which account allows for tax-free withdrawals in retirement?
   - o Traditional IRA
   - o **Roth IRA (65.4%)**
   - o 401(k)
   - o 457
   - o I don't know
5. A 15-year mortgage typically requires higher monthly payments than a 30-year mortgage but the total interest over the life of the loan will be less.
   - o **True (94.5%)**
   - o False
   - o I don't know
6. Imagine that the interest rate on your savings account is 1 percent a year and inflation is 2 percent a year. After one year, would the money in the account buy more than it does today, exactly the same or less than today?
   - o More
   - o Same
   - o **Less (90.5%)**
   - o I don't know
7. A "no-load" mutual fund is one that…
   - o Carries no fees
   - o **Carries no sales charges (13.3%)**
   - o Does not contain high-risk securities
   - o Has no limits on the period of time in which it can be bought and sold
   - o I don't know
8. A Section 529 Plan is a tax-advantaged way to save for:
   - o **College (46.4%)**
   - o Retirement
   - o Long-term health care
   - o I don't know
9. Which of the following is true about Health Savings Accounts:
   - o **You must have a high-deductible health plan in order to make contributions (11.8%)**
   - o You must have a high-deductible health plan in order to make withdrawals
   - o After age 62, you can spend the money in an HSA on a boat without a penalty
   - o HSAs provide a tax deduction when you contribute, but provide withdrawals are taxed as ordinary income
   - o I don't know
10. Which of these two funds has less oversight from government regulators?
    - o Mutual funds
    - o **Hedge funds (39.3%)**
    - o They receive the same oversight
    - o I don't know

11. Which of the following organizations insures you against your losses in the stock market?
    - o FDIC (Federal Deposit Insurance Corporation)
    - o FINRA (Financial Industry Regulatory Authority)
    - o SEC (Securities and Exchange Commission)
    - o SIPC (Securities Investor Protection Corporation)
    - o **None of the above (41.2%)**
    - o I don't know
12. If a company files for bankruptcy, which of the following securities is most at risk of becoming virtually worthless?
    - o The company's preferred stock
    - o **The company's common stock (43.6%)**
    - o The company's bonds
    - o I don't know
13. Which is the best definition of "selling short"?
    - o Selling shares of a stock shortly after buying it
    - o Selling shares of a stock before it has reached its peak
    - o Selling shares of a stock at a loss
    - o **Selling borrowed shares of a stock (22.7%)**
    - o I don't know
14. If you buy a company's stock…
    - o **You own a part of the company (81.5%)**
    - o You have lent money to the company
    - o You are liable for the company's debts
    - o The company will return your original investment to you with interest
    - o I don't know
15. If you buy a company's bond...
    - o You own a part of the company
    - o **You have lent money to the company (71.3%)**
    - o You are liable for the company's debts
    - o You can vote on shareholder resolutions
    - o I don't know
16. If interest rates rise, what will typically happen to bond prices?
    - o Rise
    - o **Fall (19.9%)**
    - o Stay the same
    - o No relationship
    - o I don't know
17. Which type of bond is the safest?
    - o **U.S. Treasury bond (76.3%)**
    - o Municipal bond
    - o Corporate bond
    - o I don't know
18. Which of the following is the best definition for a "junk bond?"
    - o **A bond that is rated as "below investment-grade" by rating agencies (39.6%)**
    - o A bond that has declined dramatically in value
    - o A bond that has defaulted
    - o A bond that is not regulated
    - o I don't know
19. Which of the following best explains why many municipal bonds pay lower yields than other government bonds?
    - o Municipal bonds are lower risk
    - o There is a greater demand for municipal bonds
    - o **Municipal bonds can be tax-free (18.5%)**
    - o I don't know
20. The principal difference between mutual fund share classes (Class A, Class B, Class C, etc.) is:
    - o The different investments each class makes
    - o **The different fees and expenses each class charges (16.1%)**
    - o The different investment advisers in charge of managing each class
    - o I don't know

Part 2 - Current Financial Planning Status, Financial Attitudes/Behaviors

21. Overall, thinking of your assets, debts and savings, how satisfied are you with your current personal financial condition? Please use a 10-point scale, where 1 means “Not At All Satisfied” and 10 means “Extremely Satisfied.”

22. When thinking of your financial investments, how willing are you to take risks? Please use a 10-point scale, where 1 means “Not At All Willing” and 10 means “Very Willing.”
23. Over the past year, how much did you spend in relation to your annual household income? Do not include unusual large purchases, such as the purchase of a new house or car, or other big investments you may have made. (*multiple choice)*
    - o Spent less than my annual household income
    - o Spent more than my annual household income
    - o Spent about the same as my annual household income
    - o Don’t know
24. In a typical month, how difficult is it for you to cover your expenses and pay all your bills? (*multiple choice)*
    - o Very difficult
    - o Somewhat difficult
    - o Not at all difficult
25. Have you set aside emergency or rainy day funds that would cover your expenses for at least 3 months, in case of sickness, job loss, economic downturn, or other emergencies? (*multiple choice)*
    - o Yes
    - o No
26. Do you currently have any dependent children? (multiple choice)
    - o Yes
    - o No
27. (*If yes*) Do you currently have any money set aside for your children’s college education? (multiple choice)
    - o Yes
    - o No
28. Have you ever tried to figure out how much you need to save for retirement? (*multiple choice)*
    - o Yes
    - o No
29. How confident are you that you could come up with $2,000 if an unexpected need arose within the next month? (*multiple choice)*
    - o I am certain I could come up with the full $2,000
    - o I could probably come up with $2,000
    - o I could probably not come up with $2,000
    - o I am certain I could not come up with $2,000
30. In the past 12 months have you obtained a copy of your credit report? (*multiple choice)*
    - o Yes
    - o No
31. In the past 12 months have you checked your credit score? (*multiple choice)*
    - o Yes
    - o No
32. Have you attended any financial planning seminars since starting your residency and/or fellowship at Washington University/University of Arizona? (*multiple choice)*
    - o Yes
    - o No
33. In the last 5 years, have you asked for any advice from a financial professional about any of the following? (*multiple choice)*
    - o Yes
        - ▪ *(If yes)* Check all areas in which you have asked for advice
            - Debt Counseling
            - Savings or investments
            - Taking out a mortgage or a loan
            - Insurance of any type
            - Tax planning
            - Estate planning
            - Asset protection
        - ▪ How did you pay the financial professional for his/her advice? (*multiple choice)*
            - o Commission on products I purchase
            - o Hourly fee regardless of type or quantity of advice
            - o Yearly fee/retainer regardless of type or quantity of advice
            - o Percentage of assets under management (AUM)
            - o Combination of above
            - o I received all of the advice for free
            - o I don't know
    - o No

34. Do you currently have any student loans? Please include all undergraduate and graduate school debt. *(multiple choice)*
    - ▪
        - $0-24,999
        - $25,00-$49,999
        - $50,00-$74,999
        - $75,000-$99,999
        - $100,000-$124,999
        - $125,000-$149,999
        - $150,000-$174,999
        - $175,000-$199,999
        - $200,000-$224,999
        - $225,000-$249,999
        - $250,000 or more
    - o No
35. Do you currently have a home loan or mortgage? If so, approximately how much do you currently owe? (*multiple choice)*
    - o Yes
        - ▪ Approximately how much do you currently owe on your mortgage? (*drop down box)*
            - $0-$49,999
            - $50,000-$99,999
            - $100,000-$149,999
            - $150,000-$199,999
            - $200,000-$249,999
            - $250,000-$299,999
            - $300,000-$349,999
            - $350,000-$399,999
            - $400,000 or more
    - o No
36. Do you currently have any credit card debt that you will not pay off at the end of the month? (*multiple choice)*
    - o Yes
        - ▪ Approximately how much credit card debt do you expect to carry over to next month? (*drop down box)*
            - $0-$999
            - $1,000-$1,999
            - $2,000-$2,999
            - $3,000-$3,999
            - $4,000-$4,999
            - $5,000-$5,999
            - $6,001-$6,999
            - $7,000-$7,999
            - $8,000-$8,999
            - $9,000-$9,999
            - $10,000 or more
    - o No
37. Do you currently have a will? (*multiple choice)*
    - o Yes
    - o No
38. Do you currently have any money saved for retirement? (*multiple choice)*
    - o Yes
        - ▪ How much do you have saved? *(drop down box)*
            - $0-$24,999
            - $25,000-$49,999
            - $50,000-$74,999
            - $75,000-$99,999
            - $100,000-$124,999
            - $125,000-$149,999
            - $150,000-$174,999
            - $175,000-$199,999
            - $200,000-$224,999
            - $225,000-$249,999
            - $250,000 or more
        - ▪ Please select all the ways you currently have money saved for retirement *(check box)*
            - Cash (checking/saving account)

- Roth IRA
- Traditional IRA
- Employer sponsored retirement plan (401k, 403b, etc)
- Pension plan
- Non-retirement investment account
- Other
    - Please specify the ‘other’ way(s) in which you are saving for retirement (*text box*)
- No

39. After residency/fellowship training, do you plan to stay in an academic/university setting (e.g. WashU), private practice settings (outpatient or hospital based), or other settings? (*drop down box)*
    - Academic/university
    - Private
    - Uncertain
    - Other
        - Please specify the ‘other’ setting in which you plan to be after completing training (*text box*)

Background/demographic questions

40. Please enter your age in years (*2 digit #)*
41. In what year did you graduate medical school (*4 digit #)*
42. Please choose your postgraduate year of training (*drop down box,* PGY 1-10 or 11+)
43. What is your department/specialty? If you are completing a fellowship, select your specialty training program (e.g. if in adult cardiology, choose internal medicine. If in pediatric urology, choose surgery). (*drop down box of all departments)*
44. What is your marital status? *(multiple choice)*
    - Married/Partner
    - Single
    - Separated
    - Divorced
    - Widow/widower
45. Please indicate the highest educational level attained by your mother *(drop down box)*
    - Some High School Or Less
    - High School Graduate
    - Specialized Business Or Technical Training (Beyond High School)
    - Some College (Incl. Associate Degree)
    - Completed College (B.A., B.S.)
    - Some Graduate Or Professional School (Required Beyond College)
    - Master's degree (M.A., M.S., M.P.H., M.B.A., etc.)
    - J.D.
    - Ph.D., Ed.D. Sci.D., Or Equivalent
    - D.D.S., D.V.M., D.P.M., Or Equivalent
    - M.D., D.O., Or Equivalent
    - M.D./Ph.D.
    - M.D., D.O., Or Equivalent plus other advanced degree (not Ph.D.)
    - I do not know
46. Please indicate the highest educational level attained by your father. *(drop down box)*
    - Some High School Or Less
    - High School Graduate
    - Specialized Business Or Technical Training (Beyond High School)
    - Some College (Incl. Associate Degree)
    - Completed College (B.A., B.S.)
    - Some Graduate Or Professional School (Required Beyond College)
    - Master's degree (M.A., M.S., M.P.H., M.B.A., etc.)
    - J.D.
    - Ph.D., Ed.D. Sci.D., Or Equivalent
    - D.D.S., D.V.M., D.P.M., Or Equivalent
    - M.D., D.O., Or Equivalent
    - M.D./Ph.D.
    - M.D., D.O., Or Equivalent plus other advanced degree (not Ph.D.)
    - I do not know

47. Please indicate your mother's occupation in the past year. *(drop down box)*
    - o Physician
    - o Dentist, Veterinarian, Optometrist, Podiatrist, Or Pharmacist
    - o Nurse
    - o Health Worker Other Than Above
    - o Lawyer Or Judge
    - o Mathematician Or Computer Scientist
    - o Natural Scientist
    - o Teacher--College/University
    - o Teacher--Other Than College/University
    - o Owner, Executive, Manager--Corporate Business, Or Small Business
    - o Technician Or Semi-Professional Not Listed Elsewhere
    - o Business Or Professional Not Listed Elsewhere
    - o Secretary
    - o Other Clerical Worker (File Clerk, Bookkeeper, Receptionist)
    - o Sales
    - o Service Worker (Police Officer, Firefighter, Other Service)
    - o Skilled Worker
    - o Unskilled Worker
    - o Farmer, Farm Manager, Farm Supervisor, Or Farm Worker
    - o Homemaker
    - o Student
    - o Other
    - o Unemployed/Unable to work
    - o Retired
    - o I do not know
    - o My mother is deceased
48. Please indicate your father's occupation in the past year. *(drop down box)*
    - o Physician
    - o Dentist, Veterinarian, Optometrist, Podiatrist, Or Pharmacist
    - o Nurse
    - o Health Worker Other Than Above
    - o Lawyer Or Judge
    - o Mathematician Or Computer Scientist
    - o Natural Scientist
    - o Teacher--College/University
    - o Teacher--Other Than College/University
    - o Owner, Executive, Manager--Corporate Business, Or Small Business
    - o Technician Or Semi-Professional Not Listed Elsewhere
    - o Business Or Professional Not Listed Elsewhere
    - o Secretary
    - o Other Clerical Worker (File Clerk, Bookkeeper, Receptionist)
    - o Sales
    - o Service Worker (Police Officer, Firefighter, Other Service)
    - o Skilled Worker
    - o Unskilled Worker
    - o Farmer, Farm Manager, Farm Supervisor, Or Farm Worker
    - o Homemaker
    - o Student
    - o Other
    - o Unemployed/Unable to work
    - o Retired
    - o I do not know
    - o My father is deceased
